# Prevalence of Depression, Anxiety, and Stress among the General Population in Saudi Arabia during Covid-19 Pandemic

**DOI:** 10.3390/ijerph17249183

**Published:** 2020-12-09

**Authors:** Hasan Saeed Alamri, Abdullah Algarni, Shehata F. Shehata, Ali Al Bshabshe, Nada N. Alshehri, Abdalla M. ALAsiri, Amjad H. Hussain, Abdulrahman Y. Alalmay, Eman A. Alshehri, Yahya Alqarni, Norah F. Saleh

**Affiliations:** 1 Department of Medicine, College of Medicine, King Khalid University, Abha 61421, Saudi Arabia; albshabshe@yahoo.com (A.A.B.); dr_nada@hotmail.co.uk (N.N.A.); 2 Ministry of Health, Abha 11176, Saudi Arabia; abaid1406@gmail.com (A.A.); abokhogmah@hotmail.com (A.M.A.); Eam.sh90@gmail.com (E.A.A.); dr.norahfayz@gmail.com (N.F.S.); 3 Department of Family and Community Medicine, College of Medicine, King Khalid University, Abha 62529, Saudi Arabia; shehatafarag@yahoo.com; 4 Biostatistics Department, High Institute of Public Health, Alexandria University, 65 Garidet St., El Horeya Rd., El Shatby, Alexandria 21526, Egypt; 5 Medical City, King Khalid University, Abha 61421, Saudi Arabia; Amjadmd2014@yahoo.com (A.H.H.); Dr.almay@yahoo.com (A.Y.A.); 6 Critical Care Medicine, National Guard Hospital, Riyadh 14611, Saudi Arabia; dr.yagarni@gmail.com

**Keywords:** coronavirus, DASS-21, psychological

## Abstract

Coronavirus disease 2019 (COVID-19) pandemic has had a significant impact on public mental health. Our objective was to assess prevalence of depression, anxiety, and stress among the general population in Saudi Arabia during this pandemic. A descriptive cross-sectional approach was used targeting all accessible populations in Saudi Arabia. Data were collected from participants using an electronic pre-structured questionnaire. Psychological impact was assessed using the Arabic version of Depression, Anxiety, and Stress Scale (DASS-21). A total of 1597 participants completed the survey. In total, 17.1% reported moderate to severe depressive symptoms; 10% reported moderate to severe anxiety symptoms; and 12% reported moderate to severe stress levels. Depression, anxiety, and stress were significantly higher among females, younger respondents, and health care providers. Depression was higher among smokers, singles, and non-working respondents. Anxiety was higher among those reporting contacts with COVID-19 positive cases, previously quarantined and those with chronic health problems. Our findings reaffirm the importance of providing appropriate knowledge and specialized interventions to promote the mental well-being of the Saudi population, paying particular attention to high-risk groups.

## 1. Introduction

Severe acute respiratory syndrome coronavirus 2 (SARS-CoV-2) is the strain of coronavirus that causes coronavirus disease 2019 (COVID-19), the highly infectious illness responsible for the COVID-19 pandemic [1]. The first cases were reported as a zoonotic transmission event in Wuhan, China, at the end of 2019 and spread to other countries, leading the World Health Organization (WHO) to declare COVID-19 a global health emergency of international concern [2,3]. More than 203 countries, areas, or territories have been affected by the virus so far, with about 16,670,063 infected and nearly 659,077 deaths reported by 28 July 2020 [4].

In Saudi Arabia, the first case was detected on 2 March 2020, after which there has been a rapid rise in cases [5]. As of 13 April 2020, educational institutes (schools and universities), commercial centers, restaurants, beaches, and resorts were closed, and a 24-h curfew has been implemented in many cities in Saudi Arabia [6]. Residents are authorized to leave for essentials, like food and medications, between 6 a.m. and 3 p.m. on the requirement that they stay within the limits of their living area, and only one passenger per vehicle is allowed [6]. Like many other countries, Saudi Arabia has suspended national and international travel, and citizens returning from abroad were placed under a mandatory 14-day quarantine [7]. The Saudi government temporarily banned Umrah pilgrimages to the holy cities of Mecca and Medina for Saudi citizens and the kingdom’s residents due to concerns over coronavirus [8].

Even though COVID-19 has emerged very recently, due to the unusual nature of this pandemic, several studies have already been accomplished to examine its psychological consequences, primarily in China and Europe [9,10,11]. Studies from China, the first affected country, suggests that the fear of this pandemic can bring about mental illness such as stress disorders, anxiety, depression, somatization, and behaviors such as increased alcohol and tobacco consumption [12]. Furthermore, the application of strict lockdown measures in that country affected many aspects of people’s lives, causing a wide variety of psychological problems, such as panic disorder, anxiety, and depression (9). A recent study using the Generalized Anxiety Disorder-7 (GAD-7) and The Center for Epidemiology Scale for Depression (CES-D) carried out in China with 7236 people showed that 20.1% of participants suffered moderate to severe depressive symptoms, and 35.1% suffered moderate to severe anxiety symptoms [13].

In Spain, another study administered the Spanish version of the Impact of Event Scale-Revised, an instrument examining psychological distress caused by a traumatic life event in terms of three symptomatic responses (avoidance, intrusion, and hyperarousal). Results from 3055 participants showed that 36.6% experienced psychological distress because of the COVID-19 pandemic. Avoidance was the most commonly cited symptom, with the psychological impact consistently higher for young people and women compared to men [14]. The Depression, Anxiety, and Stress Scale-21 (DASS-21) was administered online to 2766 respondents in Italy, revealing that 32.8% of the sample reported moderate to severe depressive symptoms; 18.7% reported moderate to severe anxiety symptoms, and 27.2% reported moderate to severe stress levels [15].

Given the paucity of research addressing the mental health impacts of COVID-19 in Saudi Arabia, the present study intends to assess the psychological impact of the national restrictive measures through a public cross-sectional survey that estimates the prevalence of depressive symptoms, anxiety symptoms, and stress during the last weeks of lockdown. Our objective was to describe the mental health implications of COVID-19 among our sample, and to identify potentially vulnerable groups or possible contributing factors targeting strategies to reduce the burden of mental health issues during the COVID-19 pandemic.

## 2. Methods

A descriptive cross-sectional approach was used targeting men and women aged 18 and over in Saudi Arabia. After obtaining permission from the Institutional ethics committee, data were collected from participants using an electronic pre-structured questionnaire. The questionnaire was uploaded online using social media platforms by the researchers and their relatives and friends between May 10 and 16 May 2020. The researchers constructed the survey tool after an intensive literature review and expert consultation. The tool was reviewed using a panel of 5 experts for content validity. Tool reliability was assessed using the study entire population with a reliability coefficient (α-Cronbach’s) of 0.89. The tool covered the following data: participants′ socio-demographic data like age, gender, residence, education, participants’ medical history, and participants hazardous practice regarding COVID-19 pandemic such as traveling abroad, contact with COVID-19 cases, and being quarantined. Psychological impact was assessed using the Arabic version of Depression, Anxiety, and Stress Scale (DASS-21), a reliable and valid measure in assessing mental health status in Arabic speakers [16]. DASS-21 is a self-report questionnaire consisting of 21 items, seven items per subscale: depression, anxiety, and stress. Patients were asked to score every item on a scale from 0 (did not apply to me at all) to 3 (applied to me very much). Sum scores were computed by adding up on the items per (sub)scale and multiplying them by 2. Sum scores for the total DASS-total scale thus range between 0 and 120, and those for each of the subscales ranged between 0 and 42. Cut-off scores of 60 and 21 were used for the total DASS score and the depression subscale, respectively. These cut-off scores were derived from a set of severity ratings, proposed by Lovibond and Lovibond [17]. Once multiplied by 2, each subscale was categorized as follows (Table 1):

### 2.1. Data Analysis

After data were extracted, it was revised, coded, and fed to statistical software IBM SPSS version 22 (SPSS, Inc. Chicago, IL, USA). All statistical analysis was completed using two-tailed tests. A p-value of less than 0.05 was statistically significant. Descriptive analysis based on frequency and percent distribution was done for all variables, including participants personal data, medical health condition, and high risk for COVID-19 practice. Scores for depression, anxiety, and stress subscales were calculated by summing up all items discrete scores. The total score for each subscale was categorized reference to the cut off points mentioned in the methodology section. Cross tabulation was used to assess the distribution of participants depression, anxiety, and stress levels by their personal and other related data. The significance of relations in cross-tabulation was tested using the Pearson chi-square test.

### 2.2. Ethical Approval

The study was conducted in accordance with the Declaration of Helsinki, and the Ethics and Research Committee of the College of Medicine of King Khalid University approved the protocol. Approval number (ECM#2020-237)—(HAPO-06-B-001)

## 3. Results

A total 1597 respondents completed the survey. They ranged in age from 18 to 75 years old, with a mean age of 36.6 ± 10.8 years; Males made up 54.5 % of the sample (*n* = 871). More than 96.1% of respondents were Saudi (*n* = 1535), and they were overwhelmingly university graduates (81.8%; *n* = 1307). Almost half (49%; *n* = 783) worked in the governmental sector, while only 12% (*n* = 188) worked in the private sector. Among all respondents, 34% (*n* = 542) worked in the health care sector, and over half (54.5%) had monthly incomes that exceeded 10,000 SR. More than two thirds (69.1%) of the sample were married, and almost half (46.6%; *n* = 547) had 3–5 children, while only 9.6% of those married had no children. Thirty-four (6.7%) female respondents were pregnant. Almost 18% (*n* = 283) of the sample were current smokers (Table 2).

Table 3 demonstrates the risk of exposure to COVID-19 among survey respondents. Approximately 8% (*n* = 118) had recently travelled, 35 (2.2%) had been exposed to a COVID-19 positive cases, and 38 (2.4%) were previously quarantined. Considering chronic health problems, most of the participants (73.4%; *n* = 1172) were healthy. The most-reported health conditions were autoimmune diseases, including asthma (7.5%; *n* = 119) followed by diabetes (7.1%; *n* = 113), hypertension with treatment (5.8%; *n* = 93), and immunosuppressive disorders (2.1%; *n* = 34).

**Figure 1:**
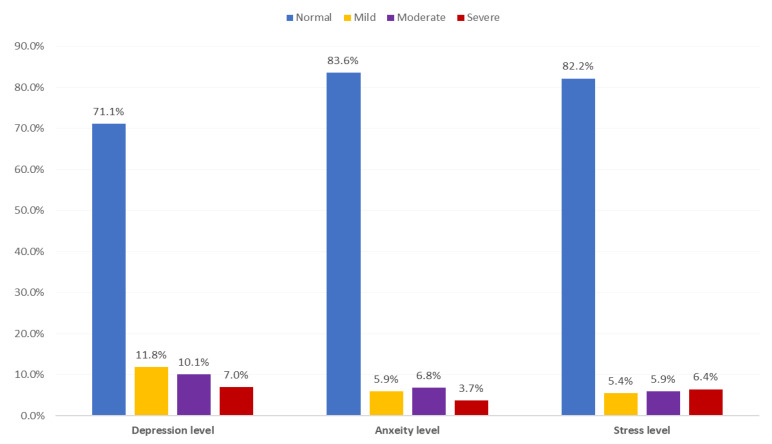
Distribution of psychological health parameters among general population in Saudi Arabia during COVID-19 pandemic, 2020.

Participants’ psychological health status during COVID-19 pandemic (Table 4 and Figure 1) illustrate that over a quarter of respondents (28.9%; *n* = 462) reported experiencing any depression, with 11.8% reporting mild symptoms, 10.1% reporting moderate symptoms and 7% reporting severe symptoms. The most-reported depressive item was feeling downhearted and blue (50.1%), followed by not experiencing any positive feelings at all (41.55%), and lack of motivation (35.1%). More than 16% of respondents experiencing anxiety (*n* = 262), with more than 10% reporting moderate to severe symptoms. The most reported anxiety factors were concerns about panic and making fool of oneself (43.5%) followed by awareness of dryness of mouth (27.5%) and unexplained fear (22.5%). Almost 18% of sample respondents reported experiencing stress, with the majority (12%) reporting moderate and severe stress symptoms. The most-reported stress items were difficulty winding down (62.6%), followed by getting agitated (49.2%) and difficulty relaxing (44%).

Table 5 shows the distribution of participants psychological health aspects by their biodemographic data. Depression, anxiety, and stress were significantly higher among females than males (33.6% vs. 25%, 19.8% vs. 13.5%, and 22.2% vs. 14.2%, respectively; *p* < 0.05). Moreover, younger respondents (<35 years) were significantly more depressed, anxious, and stressed than older respondents (>35 years) (35.6% vs. 22.2%, 20.4% vs. 12.4%, and 23% vs. 12.7%, respectively; *p* < 0.05). Non-working respondents (34.5%) were more likely to report experiencing depression than those who were working, and health care practitioner, in particular, disproportionately experienced depression, anxiety, and stress (32.7% vs. 27%, 20.1% vs. 14.5%, and 22.1% vs. 15.6%, respectively; *p* < 0.05). Single participants were significantly more depressed and anxious than others (41.5% and 25%, respectively). More current smokers reported experiencing depression and stress than others (35.7%, and 23%, respectively). Almost a quarter (24.6%) of those who had travelled abroad were experiencing stress compared to 17.3% of non-travelers (*p* = 0.047). Anxiety was significantly higher among those reporting contacts with a COVID-19 positive cases (42.9% vs. 15.8%; *p* = 0.011), and those who were previously quarantined were more anxious than others (31.6% vs. 16%, respectively; *p* = 0.011). Of those suffering from chronic health problems, 21.6% were anxious compared to 14.5% of healthy persons (*p* = 0.001). Stress was also detected among 21.4% of high-risk participants compared to 16.6% of the healthy group (*p* = 0.025).

## 4. Discussion

This study aimed to assess the psychological impact of COVID-19 pandemic on the general population of Saudi Arabia. Our survey of 1597 respondents a cross Saudi Arabia showed that 28.9% of respondents reported depressive symptoms, 16.4% reported anxiety symptoms and 17.8% reported stress symptoms. Moderate to severe features of depression, anxiety, and stress were experienced by 17.1%, 10.5%, and 12.3%, respectively. Our respondents were less likely to report experiencing anxiety and stress compared to other international studies, such as those from Iran, where the prevalence of severe anxiety was 19.1% [18], and China where moderate to severe anxiety and stress were 28.8% and 29.6 %, respectively [19]. The results of this study are similar to the results of a study conducted in Spain by Jimenez O. et al., at about the same period of time [20].

Our results suggested that being female was associated with increased depression, anxiety, and stress, which is similar to finding reported in previous studies [9,19], and similar to evidence in international literature demonstrating females tend to be more susceptible to stress and post-traumatic symptoms [20].

In the present research, young age was found to be associated with increased depression, anxiety, and stress. To date, the literature reports mixed results for this variable concerning the mental health of different age groups during the COVID-19 crisis [9,20,21,22,23,24]. Some literature in the field of disaster reveals that the elderly is particularly vulnerable to the adverse psychological sequelae of critical situations, such as post-traumatic stress disorder (PTSD) [25]. However, in agreement with our results, most of the studies have found that age constitutes a protective effect, and this trend may be explained by their greater life experience, previous disaster exposure, or having to face fewer life responsibilities [26]. Some researchers have suggested that higher anxiety amidst the younger population may be due to their greater access to information via social media, which can easily provoke stress [27]. Furthermore, it is speculated that the crisis might be presenting a much greater range of difficulties for the working-age, rather than elder age groups. For example, in addition to financial worries, it is possible that COVID-19 may be currently inducing other stressors in younger age groups that similarly impacts mental health, such as the need for both parents to telework from home while also homeschooling their children. Other factors might be important in this context, an earlier study found, for example, that older adults were more psychologically resilient than their younger counterparts [28], which might be important when it comes to reacting to the sources of stress associated with the COVID-19 pandemic. 

Being married was a protective factor against psychological suffering, as has usually been found in the literature [29,30,31]. Strangely, having children turned out to be a protective factor against psychological distress. While one might assume that being impounded at home with children may cause a higher degree of anxiety and stress, our data showed otherwise. Consistent with results from studies showing that parenthood increases subjective well-being [29,32,33], our findings likewise showed that being a parent confers a level of protection from COVID-19 related mental health issues.

In contrast to Wang C. et al., study results [19], educational level has no significant association with the psychological impact of COVID-19, a finding similar to a Chinese study [34]. One explanation is that most of the participants in the current study hold university education or higher, and they all completed the online questionnaire by themselves, indicating they have access to online sources of information, thus attenuating the impact of educational background.

The present study found an association between a history of chronic medical problems and increased anxiety and stress. These finding echoes previous studies indicating that chronic illness or a self-evaluation of poor health is associated with increased psychological distress [19,35]. A possible interpretation for this finding is that persons with a history of medical problems who also perceive their health as weak might feel more vulnerable to contracting a new disease [36].

Smoking was associated with a higher degree of depression and stress, which could be attributed to the awareness of smokers that they have a high chance of developing more medical complications if they were infected with COVID-19 because of smoking habits [37].

The results showed that health care workers were associated with increased stress anxiety, and depression. Such workers tend to have a high degree of contact with the public, and hence are at a higher likelihood of being infected; this may increase their stress levels. Moreover, this is in agreement with previous studies published recently and during the Middle East respiratory syndrome (MERS) outbreak in Saudi Arabia and studies conducted during the current COVID-19 pandemic in Singapore and India [38,39,40,41]. In addition to that, individuals who reported having contact with COVID-19 patients or history of travel abroad were found to experience higher level of depression, anxiety, and stress; and this can be attributed to different reasons, first the increased risk of contracting the disease because they may have been in contact with an infected person; secondly, he/she is worried about the health condition of his/her family, friends or colleagues.

This study had some limitations that should be considered when interpreting the data. The first one we do not have a DASS-21 baseline of pre-pandemic data and detailed pre-post analyses could not be done; hence, we cannot be sure of any increase in distress levels or if any increase (if validated) was COVID-19 related. The survey provides only a snapshot of psychological responses at a particular point in time, and a longitudinal study is required to provide information on whether the observed impact will last for more extended periods. Another limitation, the quality of cohabitation was not included in this study, while it was shown to be a key variable in the psychological impact of the participants, since its poorer quality was related to higher scores of stresses [20]. The psychological self-reported effects, anxiety, depression, and stress may not adequately represent the mental health status assessed in an interview; thus, for the outcome to be determined, prospective studies are necessary to provide more accurate data to support the need for focused public mental health strategies.

## 5. Conclusions

Depression, anxiety, and stress are prevalent among the general population during the COVID-19 pandemic lockdown in Saudi Arabia. We identified the specific subgroups of the general population at higher risk: females, those living alone during the COVID-19 pandemic lockdown, people with a history of smoking or chronic medical problems, and healthcare providers. Medical Authorities should focus on providing appropriate knowledge about the disease using appropriate methods, and specialized interventions to promote the mental well-being of the Saudi population, paying particular attention to high-risk groups. For instance, healthcare workers are known to be at a higher level of risk and thus should be prioritized when such interventions are implemented. Moreover, community mental health care should be made accessible to people who are at increased risk.

## Figures and Tables

**Table 1:** Cutoff points for DASS-21 scale.

Severity	Depression	Anxiety	Stress
Normal	0–9	0–7	0–14
Mild	10–13	8–9	15–18
Moderate	14–20	10–14	19–25
Severe	21–27	15–19	26–33
Extremely severe	28+	20+	34+

**Table 2:** Personal data of survey participants regarding psychological impact of COVID-19 on public, Saudi Arabia, 2020.

Personal Data		No	%
Gender	Male	871	54.5%
	Female	726	45.5%
Age in years	<18 years	29	1.8%
	18–25	270	16.9%
	26–35	501	31.4%
	36–45	488	30.6%
	46–55	223	14.0%
	56–65	77	4.8%
	>65 years	9	0.6%
Nationality	Non-Saudi	62	3.9%
	Saudi	1535	96.1%
Educational level	Below secondary	27	1.7%
	Secondary	263	16.5%
	University/more	1307	81.8%
Work	Not working	496	31.1%
	Governmental sector	783	49.0%
	Private sector	188	11.8%
	Military sector	130	8.1%
Health care practitioner	Yes	542	33.9%
	No	1055	66.1%
Monthly income	<5000 SR	435	27.2%
	50,00–10,000 SR	293	18.3%
	10,000–20,000 SR	587	36.8%
	>20,000 SR	282	17.7%
Marital status	Single	424	26.5%
	Married	1104	69.1%
	Divorced/widow	69	4.3%
Number of children	None	113	9.6%
	1–2	373	31.8%
	3–5	547	46.6%
	6+	140	11.9%
If female, pregnant?	Yes	34	6.7%
	No	476	93.3%
Smoking	Never smoker	1114	69.8%
	Current smoker	283	17.7%
	Ex-smoker	200	12.5%

**Table 3:** Risk of exposure to COVID-19 among survey participants.

Risk of Exposure to COVID-19		No	%
Was abroad during last three months	Yes	118	7.4%
	No	1479	92.6%
Contact with COVID-19 case during last month	Yes	35	2.2%
	No	1562	97.8%
Previously quarantined	Yes	38	2.4%
	No	1559	97.6%
Chronic health problems	None	1172	73.4%
	Autoimmune diseases including asthma	119	7.5%
	Hypertension under treatment	93	5.8%
	Chronic heart diseases	22	1.4%
	Diabetes Mellitus	113	7.1%
	Immunosuppressive disorders	34	2.1%
	Hypothyroidism	32	2.0%
	Renal disorders	5	0.3%
	Others	91	5.7%

**Table 4:** Depression, Anxiety, and Stress Scale (DASS) distribution among survey participants during COVID-19, Saudi Arabia, 2020.

Domain	Items	Did Not Apply to Me at All		Applied to Me to Some Degree, or Some of the Time		Applied to Me to a Considerable Degree or a Good Part of Time		Applied to Me Very Much or Most of the Time	
		No	%	No	%	No	%	No	%
Depression	I could not seem to experience any positive feeling at all	935	58.5%	467	29.2%	141	8.8%	54	3.4%
	I found it difficult to work up the initiative to do things	1036	64.9%	396	24.8%	101	6.3%	64	4.0%
	I felt that I had nothing to look forward to	1042	65.2%	376	23.5%	102	6.4%	77	4.8%
	I felt down hearted and blue	797	49.9%	556	34.8%	133	8.3%	111	7.0%
	I was unable to become enthusiastic about anything	1174	73.5%	302	18.9%	72	4.5%	49	3.1%
	I felt I wasn’t worth much as a person	1224	76.6%	242	15.2%	84	5.3%	47	2.9%
	I felt that life was meaningless	1170	73.3%	276	17.3%	72	4.5%	79	4.9%
Anxiety	I was aware of dryness of my mouth	1158	72.5%	351	22.0%	63	3.9%	25	1.6%
	I experienced breathing difficulty	1372	85.9%	176	11.0%	36	2.3%	13	0.8%
	I experienced trembling	1437	90.0%	133	8.3%	13	.8%	14	0.9%
	I was worried about situations in which I might panic and make a fool of myself	902	56.5%	482	30.2%	136	8.5%	77	4.8%
	I felt I was close to panic	1480	92.7%	96	6.0%	16	1.0%	5	0.3%
	I was aware of the action of my heart in the absence of physical exertion	1445	90.5%	108	6.8%	24	1.5%	20	1.3%
	I felt scared without any good reason	1238	77.5%	277	17.3%	54	3.4%	28	1.8%
Stress	I found it hard to wind down	597	37.4%	715	44.8%	199	12.5%	86	5.4%
	I tended to over-react to situations	962	60.2%	458	28.7%	129	8.1%	48	3.0%
	I felt that I was using a lot of nervous energy	957	59.9%	481	30.1%	104	6.5%	55	3.4%
	I found myself getting agitated	812	50.8%	563	35.3%	142	8.9%	80	5.0%
	I found it difficult to relax	895	56.0%	468	29.3%	142	8.9%	92	5.8%
	I was intolerant of anything that kept me from getting on with what I was doing	1066	66.8%	392	24.5%	86	5.4%	53	3.3%
	I felt that I was rather touchy	972	60.9%	470	29.4%	102	6.4%	53	3.3%

**Table 5:** Distribution of participants psychological health aspects by their biodemographic data.

Bio-Demographic Data		Depression		Anxiety		Stress	
		No	%	No	%	No	%
Gender	Male	218	25.0%	118	13.5%	124	14.2%
	Female	244	33.6%	144	19.8%	161	22.2%
*p-*Value		0.001 *		0.001 *		0.001 *	
Age in years	<35 years	285	35.6%	163	20.4%	184	23.0%
	>35 years	177	22.2%	99	12.4%	101	12.7%
*p-*Value		0.001 *		0.001 *		0.001 *	
Nationality	Non-Saudi	23	37.1%	15	24.2%	14	22.6%
	Saudi	439	28.6%	247	16.1%	271	17.7%
*p-*Value		0.148		0.091		0.321	
Educational level	Below secondary	6	22.2%	5	18.5%	2	7.4%
	Secondary	71	27.0%	37	14.1%	39	14.8%
	University/ more	385	29.5%	220	16.8%	244	18.7%
*p-*Value		0.536		0.520		0.120	
Work	Not working	171	34.5%	87	17.5%	97	19.6%
	Governmental sector	203	25.9%	126	16.1%	132	16.9%
	Private sector	51	27.1%	26	13.8%	37	19.7%
	Military sector	37	28.5%	23	17.7%	19	14.6%
*p-*Value		0.011 *		0.662		0.412	
Health care practitioner	Yes	177	32.7%	109	20.1%	120	22.1%
	No	285	27.0%	153	14.5%	165	15.6%
*p-*Value		0.019 *		0.004 *		0.001 *	
Marital status	Single	176	41.5%	89	21.0%	106	25.0%
	Married	266	24.1%	155	14.0%	165	14.9%
	Divorced/ widow	20	29.0%	18	26.1%	14	20.3%
*p-*Value		0.001 *		0.001 *		0.001 *	
Number of children	None	30	26.5%	21	18.6%	15	13.3%
	1–2	117	31.4%	73	19.6%	83	22.3%
	3–5	124	22.7%	70	12.8%	75	13.7%
	6+	15	10.7%	9	6.4%	6	4.3%
*p-*Value		0.001 *		0.001 *		0.001 *	
If female, pregnant?	Yes	14	41.2%	7	20.6%	10	29.4%
	No	138	29.0%	88	18.5%	90	18.9%
*p-*Value		0.133		0.761		0.136	
Smoking	Never smoker	311	27.9%	181	16.2%	190	17.1%
	Current smoker	101	35.7%	52	18.4%	65	23.0%
	Ex-smoker	50	25.0%	29	14.5%	30	15.0%
*p-*Value		0.015 *		0.509		0.036 *	
Was abroad during last three months	Yes	41	34.7%	26	22.0%	29	24.6%
	No	421	28.5%	236	16.0%	256	17.3%
*p-*Value		0.148		0.086		0.047 *	
Contact with COVID-19 case during last month	Yes	15	42.9%	15	42.9%	9	25.7%
	No	447	28.6%	247	15.8%	276	17.7%
*p-*Value		0.066		0.001 *		0.219	
Previously quarantined	Yes	11	28.9%	12	31.6%	5	13.2%
	No	451	28.9%	250	16.0%	280	18.0%
*p-*Value		0.998		0.011 *		0.445	
Chronic health problems	None	326	27.8%	170	14.5%	194	16.6%
	High risk health condition	136	32.0%	92	21.6%	91	21.4%
*p-*Value		0.103		0.001 *		0.025 *	

P: Pearson X^2^ test; * *p* < 0.05 (significant).
